# What “causal cognition” might mean

**DOI:** 10.3389/fpsyg.2014.01204

**Published:** 2014-10-22

**Authors:** D. B. Kronenfeld

**Affiliations:** Anthropology, University of California, RiversideRiverside, CA, USA

**Keywords:** collective cognition, culture, kinds of causality, agency, cultural models, action plans

As something of an outsider to the study of “causal cognition,” I want briefly to query what it might be taken to mean in general—outside of any particular disciplinary understanding. Next, from that perspective I look at some empirical approaches to selecting problem-relevant senses of causality, and senses in which cognition might be seen as causal. I then turn, at more length, to the nature and significance of collective causal cognition, including the cultural models type.

## The subjective perspective—our cognition of causality

Our default senses of what we mean by causality vary from one another. My own personal default is that causality is human and individual (vs. collective), and that culture provides expectations regarding what kinds of causality are understood to work in the world, what kinds apply to “all people” and what kinds (physical, psychological, social, etc.) apply in what form to other individuals.

One can, also, separately—as an outside observer—consider the causal processes that one sees working on or in a group—from mob behavior on up. Some of this attribution of causal processes seems universal while other seems culture-specific, or, even, more individual.

### A range of meanings and measures

But note that the issue depends on what one means by “cause” and by “cognition”—and thus on where one's interests lie. There exist various (well-known!) kinds of “causes” in addition to the efficient (or active) causes I opened with—such as final causes, indirect causes, enabling conditions, and so forth. “Cognition” can range from individual knowledge without any active decision-making element, through more broadly defined knowledge that includes an individual's potential action plans, to collectively held knowledge including appropriate collective action. Alternatively, “cognition” can go to the root of action, as in the “flight/fight response,” where a uniform physiological response in the brain can be interpreted as fear (leading to flight) or aggression (leading to fight)—depending on the situation/context and on one's prior experience. One's modeling of the mental states of other beings—“theory of mind”—also represents a potentially causative cognitive activity.

## Empirical approaches

One way to approach the general issue of causal cognition might be to take everything that is needed for a simulation of some action/event (such as, for example, Schank and Abelson, 1977 restaurant simulation) and then see what of that is cognitive—and in what sense. But I suspect that the answer might be overwhelming in both its breadth and its length!

More sensible, perhaps, is for one to consider why in particular one is asking about “causal cognition” and see what speaks to that particular instance or version.

As an ethnographer one can turn to people's everyday default senses of what they mean when they speak of the “cause” of some activity or situation. Based on Evans-Pritchard's classic Zande example (Evans-Pritchard, 1937; pp. 69–70), one might ask “why did the corn crib fall on Uncle Joe?” The answer is the answerer's sense of what “caused” it to fall. That is, the answer is an instance of cognition about causality. If the view is widely shared within the culture, we have an instance of culturally shared cognition, and if people in the culture act on the answer (based on shared and accepted views of, say, crime and action), then we have cognitively caused social action.

If, in the Zande example, an actor argues for a particular response—based on what happened and on those shared and accepted views of crime and action—then we have an instance of individual cognitive causality (since her understanding has led to her arguing a case). Evans-Pritchard says the typical Zande answer would be a statement about whose witchcraft triggered the collapse. And a social witchcraft settlement process might be initiated. The process would involve culturally-based understandings of what kinds of events trigger witchcraft accusations.

In my American culture the answer would be “because the crib was rotten from a termite infestation”—a material state answer. And possibly the polity might enact stricter corn crib inspection standards, or punish the builder for faulty construction practices. Evan-Pritchard's Zande are aware of the risk posed by a termite infestation, but are more concerned with why the collapse happened *particularly when Joe was there*. We, on the other hand, tend to dismiss the timing question with “It's chance” or “Shit happens” responses.

But, the range of “causes” is still far from exhausted. We can come up with a raft of enabling causes. For example, why was the corn crib built (in that place, and so insubstantially)? Why was Uncle Joe sleeping there? And, for that matter, why were the Azande people there (vs. somewhere else) growing corn that had to be stored in that manner?

## Collective cognition and causality

Nadel (1952) provides a different kind of example of the interaction of witchcraft accusations with causally relevant cognitively based collective social structures (formal age grades and the power—including property—which goes with each) and demographic factors in two East African societies. In both societies, when men enter the senior grade they are supposed to turn over their political power and major economic goods to their heirs. In the society with few age grades, the turnover takes place while the new seniors are still relatively young, vigorous, and ambitious. These “rising seniors”—much resenting the pressure of their heirs to move on and make room for the next class—try to drag out the process, which causes resentment among the class of their heirs, which leads to accusations of witchcraft against the seniors (for trying to hold the heirs back). In the society with a greater number of grades, the seniors are older when the turnover takes place, and more ready to move on, and the class of heirs is in a middle-age grade that entails a significant societal role, and so they are much less apt to be resentful. In Nadel's examples, it is culturally standardized knowledge about the consequences of age grade membership which produces (“causes”) the incidence of witchcraft accusations.

Collective cognition that involves an action is necessarily causative because it is only the collective knowledge that makes the action efficacious. That is, the products of actions such as marriage (see below) don't exist unless relevant communities recognize them.

Collective action of many sorts depends on differentially shared and overlapping knowledge, knowledge that involves shared goals, shared procedures and rules, shared expectations about likely actions, and insightful interpersonal knowledge. Mundane examples can be seen in the behavior of a well-organized soccer or basketball team. Effective offense depends not just on organized plays, but even more on knowledge of teammates' personal characteristics in the context of a play and of opponents' likely responses. Successful defense depends on a shared but shifting dynamic understanding of the playing space and the flow of action in it—not just where the ball is or who has it, but where it's likely to go and how it's going to get there.

A similar kind of collective knowledge was pointed out by Romer (1984) in connection with the coordination among members of an ancient Egyptian work group implied by their production of art forms in which a single line flows as if carved in a continuous act by a single hand—where size and material would make execution by a single hand impossible. Classical European painters' ateliers have sometimes exhibited that collective unity.

Marriage is an example of collective cognition that can cause substantial effects. Marriage can “cause” property ownership (as in “Why does she own that house?” “Because her husband bought it, and it's joint property”). People are only married—with the resulting legal and social concomitants—if they are known to be married—even if that knowledge, in their culture, presumes some efficacious words or ritual. Much of kinship, in effect, depends similarly on knowledge—except that sometimes DNA can be appealed to. Inheritance is an example of collective cognitive causality—not just its reliance on kinship but for the rules that members of the culture have defined which specify who gets a dead person's stuff and, sometimes, social and political role.

Cultural models (as in Kronenfeld, 2008 and see Bennardo and Kronenfeld, 2011) are one particular kind of collective cognitive system that can be indirectly causal. They don't directly make things happen, but in a given situation they do provide individuals with models for how they might act in a given situation.

Other apparent examples of collective cognitive causality include joint tasks by a collection of people where none of the participants know the full plan or system and where there exist no explicit written plans. Examples of such tasks include Hutchins' (1994) account of how an aircraft carrier is actually navigated inside an enclosed bay, Gatewood and Lowe's (2008) account of the nature and operation of credit unions, and my own (Kronenfeld, 2011, pp. 575–576; 2014, p. 85) example of house construction.

In Hutchins' example, it is sailors' individual knowledge of their own specific roles—including how their roles link with those they immediately connect with—that allows their behavior to fit into a patterned process. The process is kept aligned with the ultimate navigational task (including interrelating the ship's location and speed relative to the shoreline, water depth, other ships, and target dock) by someone who puts the products of the sailors' action sequence into a format that translates into the Bridge's understanding of the task and which is used as the basis of instructions to the helm and engine room—where timely execution is needed to prevent crashes and cope with surprise emergencies.

In Gatewood's example, we see that no one in the organization (not directors, officers, staff, or customers) held full knowledge of the goals, organization, and operation of a credit union, and that this information was nowhere completely written out. We see that—as it turned out—somewhat divergent views were held (by people in the different positions) of why credit unions existed, what they were useful for, and how they operated. Here, the unifying shaping comes via customer's satisfaction and usage in response to staff actions and financial offerings as guided by officers.

In my example I examined the roles involved in a small construction job—adding rooms onto an existing house in California. These roles include the owner (who commissions and pays for the work), an engineer who produces the plans (incorporating building code standards), the people who do the constructing, and the city inspectors who check for code compliance. Construction roles include the contractor who oversees the job, the carpenters, electricians, plumbers, floor installers, wallboard installers, painters, roofers, appliance installers, and so forth who do the actual work. The construction people know their own jobs through some combination of formal training and experience, and this knowledge includes how their roles interact with neighboring roles—thus they typically have some knowledge and experience of the work of these neighboring roles. Since this is a small job, and the level of expertise required for some of these roles is not too high, it is not uncommon for one person to fill several of the roles—depending on that person's training and experience. Commonly, the contractor will have started as a carpenter, and often carpenter/contractors have some experience with simple electrical work and plumbing, and so may or may not hire experts to do such work, depending on availability and price. The engineer's plans are never detailed enough to anticipate all contingencies, and so much of the detail is decided on the fly—sometimes in consultation with the owner (who may or have only limited knowledge). No one knows all that is needed for the job. Typically, the expert (relevant to a particular problem) makes decisions based on his or her knowledge, and in consultation with others directly affected; but both owners and city code enforcers play major roles.

## Conclusion

These examples illustrate how (successful) collective action depends on systems of collective knowledge and on individual possession of relevant parts of that collective knowledge. The collective project cannot take place without both collective and individual knowledge, and so the knowledge (i.e., cognition) has to be considered causative.

Finally, an understanding of even individual “causal cognition” requires attention to default understandings which often are culturally based.

### Conflict of interest statement

The author declares that the research was conducted in the absence of any commercial or financial relationships that could be construed as a potential conflict of interest.
